# Advancing CAR T-cell therapy for chronic lymphocytic leukemia: exploring resistance mechanisms and the innovative strategies to overcome them

**DOI:** 10.20517/cdr.2023.100

**Published:** 2024-05-14

**Authors:** Azra Borogovac, Tanya Siddiqi

**Affiliations:** City of Hope, Department of Hematology and Hematopoietic Cell Transplantation, Lennar Foundation Cancer Center, Irvine, CA 92618, USA.

**Keywords:** CAR T-cells, CLL, resistance mechanisms, allogeneic CART

## Abstract

Chimeric antigen receptor (CAR) T-cell therapy has ushered in substantial advancements in the management of various B-cell malignancies. However, its integration into chronic lymphocytic leukemia (CLL) treatment has been challenging, attributed largely to the development of very effective chemo-free alternatives. Additionally, CAR T-cell responses in CLL have not been as high as in other B-cell lymphomas or leukemias. However, a critical void exists in therapeutic options for patients with high-risk diseases who are resistant to the current CLL therapies, underscoring the urgency for adoptive immunotherapies in these patients. The diminished CAR T-cell efficacy within CLL can be traced to factors such as compromised T-cell fitness due to persistent antigenic stimulation inherent to CLL. Resistance mechanisms encompass tumor-related factors like antigen escape, CAR T-cell-intrinsic factors like T-cell exhaustion, and a suppressive tumor microenvironment (TME). New strategies to combat CAR T-cell resistance include the concurrent administration of therapies that augment CAR T-cell endurance and function, as well as the engineering of novel CAR T-cells targeting different antigens. Moreover, the concept of “armored” CAR T-cells, armed with transgenic modulators to modify both CAR T-cell function and the tumor milieu, is gaining traction. Beyond this, the development of readily available, allogeneic CAR T-cells and natural killer (NK) cells presents a promising countermeasure to innate T-cell defects in CLL patients. In this review, we explore the role of CAR T-cell therapy in CLL, the intricate tapestry of resistance mechanisms, and the pioneering methods studied to overcome resistance.

## INTRODUCTION

The successful development of genetically engineered chimeric antigen receptor (CAR) T-cell therapy has led to remarkable progress in the treatment of hematological malignancies^[1,2]^. Trials utilizing CAR T-cell therapy have demonstrated impressive overall response rates (ORR) of more than 80% in the treatment of relapsed/refractory (r/r) B-cell acute lymphoblastic leukemia (B-ALL), diffuse large B-cell lymphoma (DLBCL), and mantle cell lymphoma (MCL) and provide a potential for cure in 30%-40% of patients with DLBCL^[3]^. However, there has been less enthusiasm for CAR T-cells in chronic lymphocytic leukemia (CLL) than in other non-Hodgkin lymphomas (NHL) for several reasons. For one, the clinical course of CLL is generally insidious, and asymptomatic patients may go years without needing therapy. The median age for diagnosis is 70 years, and CAR T-cell therapy may not be appropriate for some elderly patients. However, real-world analysis of CAR T-cell therapy in older patients showed acceptable efficacy and safety even in individuals aged 75 years or older^[4]^. When treatment is indicated, effective, chemotherapy-free options such as Bruton tyrosine kinase (BTK) and B-cell lymphoma-2 (BCL2) inhibitors are preferred^[5,6]^. Additionally, CAR T-cell therapy has demonstrated less reliable ORR in larger studies ranging from 43%-79%, with much lower complete responses (CR)^[7-9]^. Nevertheless, treatment options remain limited in patients who are refractory to both BTKi and BCL2 inhibitors, particularly in those who have high-risk cytogenetics such as complex karyotype, unmuted immunoglobulin heavy chain gene mutation (IGHV), 17p deletion, and TP53 mutation^[10,11]^. In this setting, CAR T-cell therapy shows great promise, particularly in the challenging multi-agent refractory, high-risk population. Encouraging data from the TRANSCEND CLL 004 trial^[9]^ using the CD19 CAR T-cell product, lisocabtagene maraleucel (liso-cel), has resulted in the first Food and Drug Administration (FDA) approval for CAR T-cell therapy in CLL. In March 2024, liso-cel was approved for the treatment of CLL patients who are relapsed or refractory to at least two prior lines including BTK and BCL2 inhibitors. This marks a significant milestone in the use of CAR T-cells in CLL, yet ongoing research is needed to further improve patient outcomes. This review will discuss current data using CAR T-cell therapy in CLL, possible resistance mechanisms, and ongoing strategies to augment their safety and efficacy.

## THE EVOLUTION OF CAR T-CELLS

**Figure 1:**
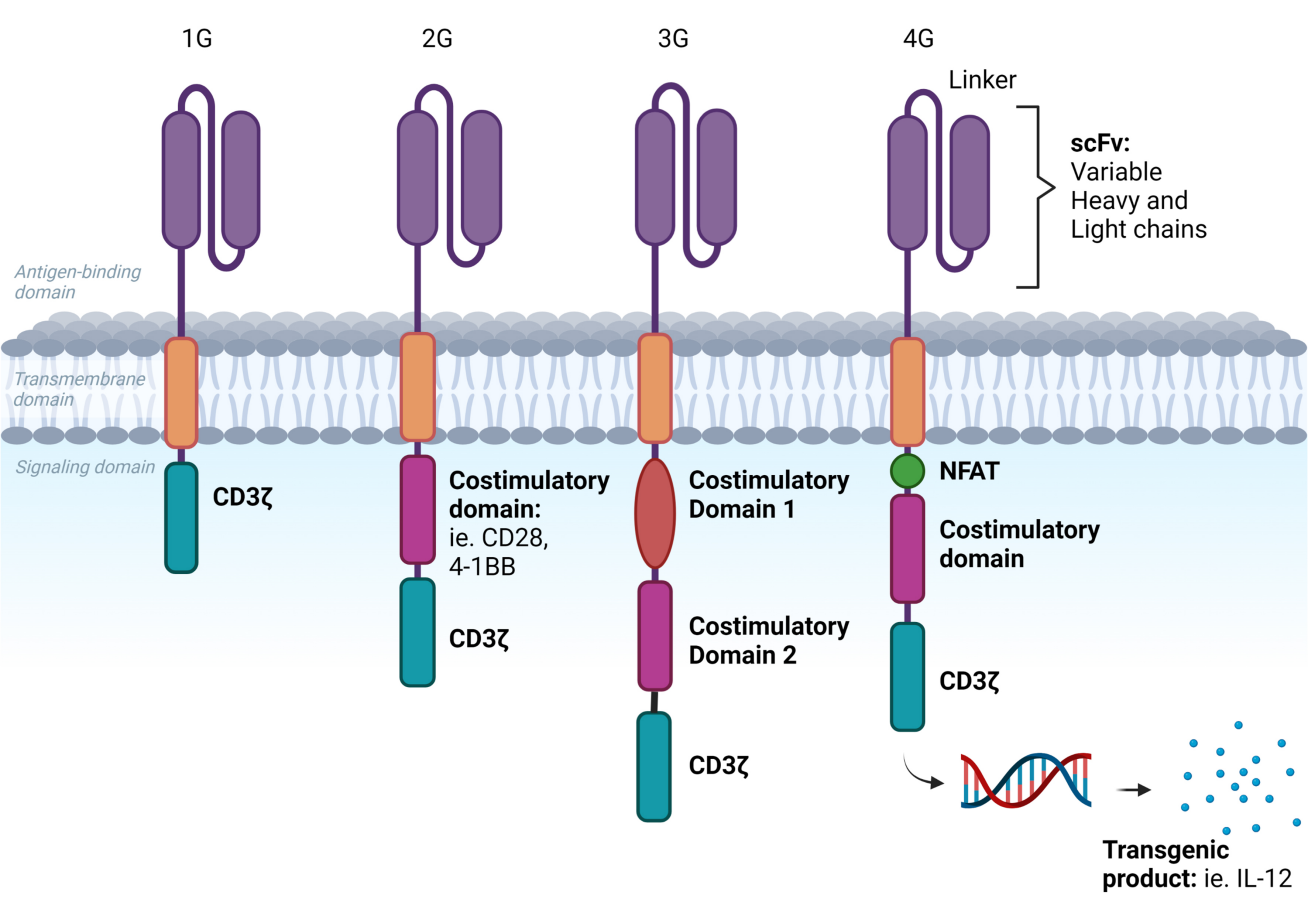
Structure of the 4 generations of CAR T-cells. First-generation (1G) CARs contain an antigen recognition domain, scFv with a variable heavy and light chain connected by a linker, a transmembrane domain, and a signaling domain (CD3ζ). Second-generation (2G) CARs are similar to 1G with the addition of a costimulatory domain such as CD28 or 4-1BB. Third-generation (3G) CARs are the same as the 2G with the addition of a second costimulatory domain. Fourth-generation (4G) CARs, also known as “armored” CARs, also produce NFATs, which release a transgenic product (such as the cytokine IL-12) that can further regulate the CAR T-cell function and influence the tumor microenvironment. CAR: Chimeric antigen receptor; scFv: single-chain variable fragment; NFATs: nuclear factor of activated T-cells; IL: interleukin.

CARs are receptor proteins that are engineered on immune cells, most commonly T-cells, allowing them to target specific antigens. CARs can be constructed on a patient’s own T-cells (autologous CARs) or on healthy donor T-cells (allogeneic CARs). CARs contain an extracellular domain with an antigen-specific single-chain variable fragment (scFv), a transmembrane domain, and an intracellular signaling domain^[2]^. First-generation CARs that harbored only a CD3ζ chain were limited in promoting T-cell activation and expansion^[12,13]^. The second-generation CARs also included a costimulatory domain, most notably CD28 or 4-1BB, which enhanced CAR T-cell expansion, cytokine production, and cell survival^[14]^. Second-generation CAR T-cells are the predominant CARs used currently in all the FDA-approved products. Third-generation CARs include multiple costimulatory domains, potentially leading to even better expansion and persistence. Lastly, fourth-generation CARs have a transgenic “payload”, also known as T-cells Redirected towards Universal Cytokine Killing (TRUCKs), which include additional anti-tumor activity such as inducible cytokine production, such as IL-12, IL-15, IL-18, and IL-23 or stimulatory ligands to influence the local tumor microenvironment (TME) and reduce systemic toxicity^[15-20]^. Figure 1 illustrates the four different generations of CAR T-cells. To date, the FDA has approved 4 CD19-directed CAR T-cell products for B-cell malignancies, including axicabtagene ciloleucel (axi-cel) and brexucabtagene autoleucel (brex-cel) both harboring a CD28 costimulatory domain, and liso-cel and tisagenlecleucel (tisa-cel) harboring a 4-1BB costimulatory domain. In addition to variances in the CAR structure, manufacturing differences may also be important in different types of lymphoma. For example, although brex-cel and axi-cel are the same product, brex-cel undergoes an additional T-cell selection step during manufacturing. This step aims to remove contamination from circulating leukemic cell involvement, which is more frequently seen in MCL and CLL than other NHL types^[21]^. Additionally, the liso-cel product uniquely processes and manufactures CD4:CD8 CAR T-cells in a 1:1 ratio. Consequently, despite potentially heterogeneous starting materials, this approach minimizes variability in cell frequencies and enhances T-cell purity through T-cell-specific activation and expansion^[9]^. As CAR constructs evolve and manufacturing processes refine, CAR T-cell therapy is poised to advance in both safety and effectiveness, ultimately becoming more widely accessible.

## CURRENT EFFICACY AND SAFETY OF CAR T-CELLS IN CLL

One of the earliest reports of successful CAR T-cells use in B-cell malignancies was reported in 2 r/r CLL patients who achieved a CR with tisa-cel^[22]^. In a recent update, these patients still have ongoing minimal residual disease (MRD) negative remissions a decade later with persistent circulating CAR T-cells^[23]^. Since that initial account, however, the use of several CD19 CAR T-cell products has been reported in CLL with varying success rates. In some studies, response rates were particularly high. For instance, Turtle *et al.* presented a study involving 24 CLL patients who had previously been treated with ibrutinib exposure and subsequently received CD19 CAR-T cell therapy^[24]^. Almost all the patients had high-risk disease with a median of 5 prior lines of therapy, 96% with complex karyotype and/or del17p and 5 patients with Richter’s transformation. The ORR by imaging criteria was 75%, and 88% of patients with bone marrow disease had clearance of their CLL cells^[24]^. Cappell *et al.* reported findings on the long-term outcomes of using the axi-cel product, which involved 7 heavily pretreated CLL patients, who had an 88% ORR and 63% CR rate. The duration of ongoing response was 82 months, with 50% responding beyond 3 years^[25]^.

Subsequent studies have depicted lower response rates; however, they repeatedly illustrate that patients who achieve a CR sustain long-term durable responses. Outcomes in 42 CLL patients randomized to receive low (5 × 10^7^ cells/kg) *vs.* high-dose (5 × 10^8^ cells/kg) CD19 CAR T-cells^[8]^ demonstrated CR and ORR of 28% and 44% at 4 weeks, respectively. The progression-free survival (PFS) and overall survival (OS) were prolonged in those who achieved a CR (40.2 months PFS, not reached OS) *vs.* those who have not achieved a CR (1-month PFS, 64 months OS). There was no statistical difference based on dose level^[8]^. More recently, Siddiqi *et al.* reported the results from the Phase 1/2 TRANSCEND CLL 004 trial, one of the largest studies utilizing liso-cel in 117 heavily pretreated patients, all with BTKi failure and 70 also with prior venetoclax. The ORR and CR rates were 43% and 18%, respectively; however, undetectable MRD was reached in 59% based on bone marrow IGH sequencing (at 1 × 10^-5^ sensitivity). These rates were similar for the 30 patients with 17p deletion or TP53 mutation with ORR of 47% and CR rates of 23%. The median PFS and OS were 11.9 and 30.3 months, respectively. However, among those with undetectable MRD, the median PFS was 26.2 *vs.* 2.8 months in those with detectable MRD. The rate of grade 3 CRS was 9% and grade 3 neurotoxicity was 18%, with only one grade 4 neurotoxicity^[9]^. The 24-month follow-up for this study was recently reported and demonstrated the median duration of response was 35.3 months and the median duration of CR or incomplete CR was not reached^[26]^. This study underscores the potential of MRD to more accurately assess deeper responses compared to traditional imaging-based response criteria in CLL. Two recent reviews have compiled a comprehensive list of CAR T-cell therapy studies in CLL^[27,28]^.

Lastly, the use of CAR T-cell therapy in patients with Richter Transformation (RT) is not well defined, mostly due to the rarity of RT^[29]^. A multi-institutional retrospective study was recently reported, which included 62 patients with RT who received CD19 CAR T-cells^[30]^. The ORR was 65% and CR was 47% after a median follow-up of 24.1 months, with a median PFS of 4.7 months and OS 8.5 months. However, the median duration of response was not reached among those who achieved a CR and only 2.3 months among those with a PR^[30]^. There is currently an ongoing Phase 2 study using brex-cel in rare B-cell malignancies including r/r RT (ZUMA-25; NCT05537766). Despite varying response rates, these studies highlight CAR T-cell therapy’s potential as a viable treatment option, even offering long-term durable remissions for some patients with limited alternatives.

## PROPOSED RESISTANCE MECHANISMS IN CLL

**Figure 2:**
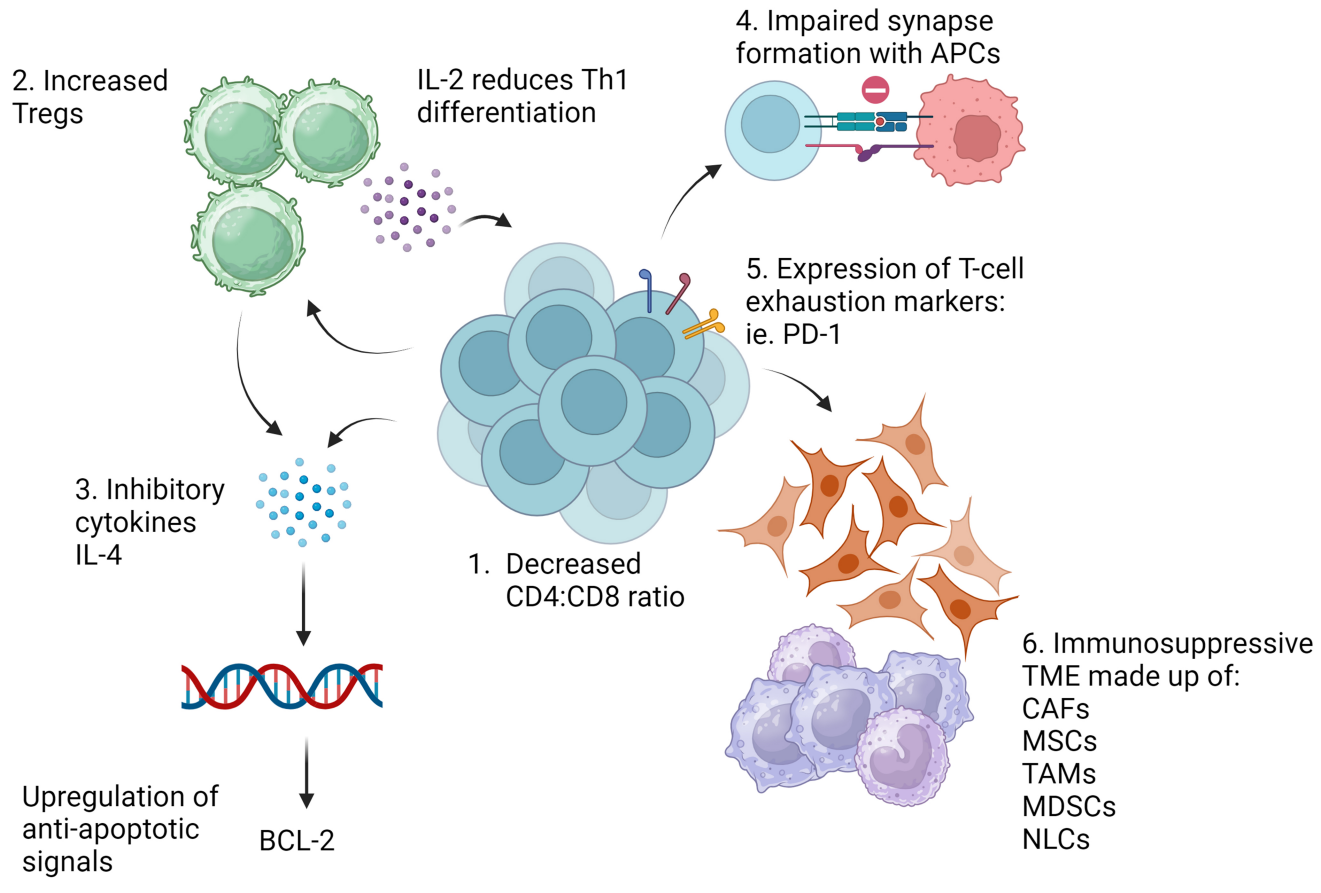
Mechanisms of impaired T-cells and immunosuppressive environment in CLL. (1) T-cells from patients with CLL demonstrate a diminished CD4 to CD8 ratio and increased numbers of Tregs. (2) Tregs, in turn, further cause increased IL-2 production, reducing Th1 differentiation. (3) Increased expression of inhibitory cytokines such as IL-4 causes upregulation of the anti-apoptotic signal, BCL2. (4) T-cells have impaired synapse formation with APCs from impaired actin polymerization. (5) Increased expression of T-cell exhaustion markers such as PD-1. (6) An immunosuppressive environment composed of multiple inhibitory cells further diminishes T-cell function and supports CLL cell survival. CLL: Chronic lymphocytic leukemia; Treg: T-regulatory cell; IL: interleukin; Th1: T-helper type 1; BCL2: B-cell ligand-2; APC: antigen-presenting cell; PD-1: programmed death-1; TME: tumor microenvironment; CAF: cancer-associated fibroblast; MSCs: mesenchymal stromal cells; TAMs: tumor-associated macrophages; MDSCs: monocyte-derived suppressor cells; NLCs: nurse-like cells.

**Figure 3:**
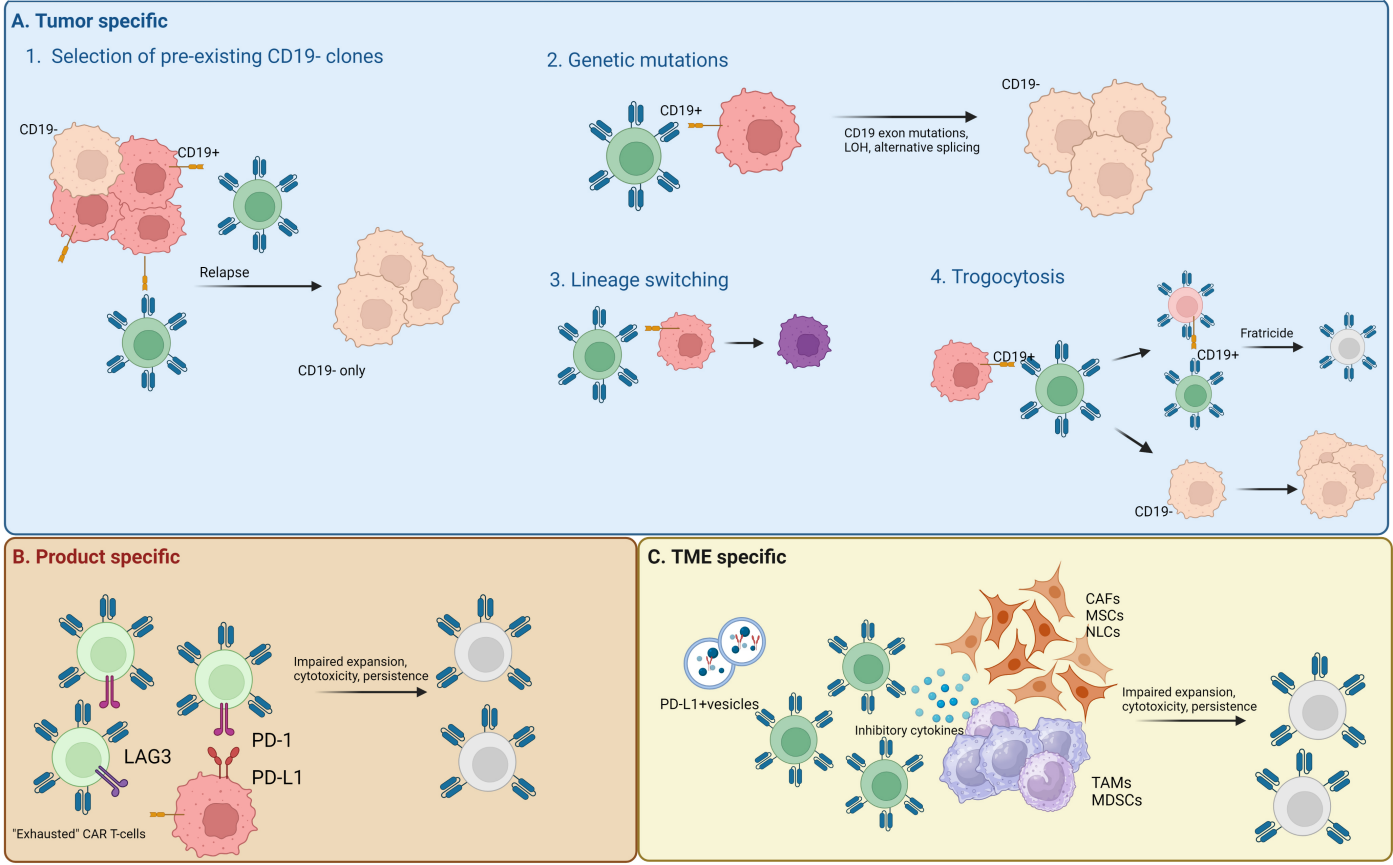
Resistance mechanisms. (A) Tumor-specific mechanisms are mostly due to selective pressure and include (1) proliferation of existing antigen-negative clones; (2) development of new genetic mutations within the antigen gene locus, loss of heterogeneity, or alternative splicing; (3) lineage switching, whereas the primary neoplastic cell becomes another type of cell (i.e., from lymphoid to myeloid); and (4) trogocytosis where the antigen of interest transfers to the CAR T-cell, leading to CAR T-cells killing each other (“fratricide”), and proliferation of the antigen-negative neoplastic cells; (B) CAR T-specific mechanisms include having CAR T-cells that have exhaustion phenotypes (i.e., expression of PD-1, LAG-3), which leads to impaired expansion, cytotoxicity, and persistence; (C) TME-specific mechanisms are due to immunosuppressive cells, inhibitory cytokines, and vesicles that protect CLL cells and cause impaired CAR T-cell function*.* CAR: Chimeric antigen receptor; PD-1: programmed death-1; LAG-3: lymphocyte-activation gene 3; TME: tumor microenvironment; CLL: chronic lymphocytic leukemia; LOH: loss of heterogeneity; PD-L1: program death ligand 1; CAF: cancer-associated fibroblast; MSCs: mesenchymal stromal cells; NLCs: nurse-like cells; TAMs: tumor-associated macrophages; MDSCs: monocyte-derived suppressor cells.

The reason for lower responses observed with CAR T-cell therapy in CLL compared to other B-cell malignancies is not completely understood, but two predominant hypotheses proposed include impaired T-cell fitness and an immunosuppressive TME [Figure 2]. These biological factors may contribute to various mechanisms of CAR T-cell resistance, including tumor-specific changes such as antigen loss or down-regulation, product-related causes, and alterations in the TME [Figure 3].

### Tumor-specific resistance mechanisms

Studies in other B-cell malignancies have shed light on antigen escape as an important mechanism of CAR T-cell resistance^[31-34]^. In both ELIANA and ZUMA-1 trials, relapse rates without CD19 expression were approximately 25% in B-ALL and 28% in DLBCL^[35,36]^. CD19 loss is thought to be caused by selective pressure and allows the tumor to evade detection of the circulating CD19-specific CAR T-cells. Multiple studies have demonstrated the expansion of CD19-negative pre-existing clones in samples collected before CAR T-cell infusion that subsequently become the dominant clone after relapse^[36-38]^. Additionally, genetic mutations, loss of heterozygosity, and alternative splicing of CD19 have been implicated in relapse^[32,38,39]^ [Figure 3]. Lineage switching, wherein a clonally related malignancy of a different cell type emerges, has also been observed. An early example of this was described in a patient with Richter transformation who received CAR T-cells and later developed plasmablastic lymphoma with identical clonal *IGH* gene rearrangement and TP53 mutation^[40]^. Trogocytosis, another intriguing mechanism, involves the transfer of the CD19 antigen to the CAR-T cell, thereby reducing antigen density on the tumors and ultimately leading to self-killing or fratricide of the CAR T-cells^[41]^. Although many of these mechanisms have yet to be described in CLL, understanding potential pathways of antigen escape will inform strategies for overcoming resistance.

### Product-specific resistance stemming from exhausted T-cells in CLL

In patients with CLL, their T-cells display an exhausted phenotype from continual antigen stimulation, resulting in poor expansion, persistence, and function^[42-44]^. Early studies have demonstrated elevated CD8+ expression and altered CD4:CD8 ratio^[45]^, leading to an activated phenotype with a shift toward a terminally differentiated effector-memory subtype^[46]^. Both CD4+ and CD8+ T-cells in CLL patients express high levels of interleukin-4 (IL-4)^[47]^, upregulating the anti-apoptotic signal, BCL-2[^48^]. Additionally, CLL patients have increased regulatory T-cells (Tregs) that have decreased responses to tumor antigens. Tregs also lead to increased IL-2 secretion, decreasing T-helper cell type 1 (Th1) differentiation^[49,50]^. Riches *et al.* demonstrated high expression of T-cell exhaustion markers CD244, CD160, and programmed death-1 (PD-1) in T-cells from patients with CLL. Overall, these T-cells had diminished expansion and cytotoxicity with defects in granzyme packaging into vesicles and nonpolarized degranulation^[51]^. There is also evidence of impaired synapse formation between both CD4+ and CD8+ T-cells and antigen-presenting cells, including CLL malignant cells^[52,53]^.

Exhausted T-cells collected for CAR T-cell manufacturing can ultimately lead to inadequate CAR T-cell proliferation, cell killing, and persistence, all crucial factors in resistance. Mueller *et al.* studied the kinetics of tisa-cel CAR T-cells in 61 patients with B-ALL and 42 patients with CLL^[54]^. In the CLL cohort, those with a CR (*n* = 8 patients) had a longer persistence of 352 days *vs.* 176 days in those with PR (*n* = 7) and 28.5 days in those who had progressive disease (*n* = 22)^[54]^. Nevertheless, expansion was higher in the patients who achieved a PR than CR. This observation suggests that more than just antigen recognition and T-cell expansion is needed to induce a CR, and that T-cell persistence also plays a pivotal role. Hoffmann *et al.* demonstrated diminished expansion of CD4+ naïve T-cells, and higher PD-1 expression in CAR T-cells from CLL patients compared to normal controls^[55]^. Patients with CLL that have increased expression of exhaustion markers such as PD-1, T-cell immunoglobulin and mucin domain 3 (TIM-3), and lymphocyte-activation gene 3 (LAG-3)^[56]^ show diminished response to CAR T-cell therapy.

The type of costimulatory domain may also be important. While the CD28 costimulatory domain enhances an early proliferative response, it may also augment early exhaustion, whereas the 4-1BB costimulatory domain may lead to more durable persistence^[57,58]^. Higher disease volume or tumor burden also predicted poor outcomes in both B-ALL^[54]^ and NHL^[59,60]^. In CLL, extramedullary disease, such as in the liver, spleen, and lymph nodes, was demonstrated to resolve more slowly than CLL in blood^[61]^. The underlying reason for this is uncertain, but it may be due to an immunosuppressive TME within bulky tumors that leads to increased interferon signaling, immune dysregulation, and CAR T-cell exhaustion^[62]^.

### CLL patients exhibit an immunosuppressive tumor microenvironment

The TME is increasingly becoming a fascinating area of research and is recognized as an important influence in CAR T-cell resistance. CLL patients have an immunosuppressive TME characterized by cancer-associated fibroblasts (CAFs)^[63]^, tumor-associated macrophages (TAMs)^[64]^, blood-derived nurse-like cells (NLC)^[65]^, monocytic myeloid derived suppressor cells (M-MDSCs)^[66]^, and abundance of CLL-derived extracellular vesicles enriched with co-inhibitory signals such as PD-1^[67]^ [Figure 2].

Paggetti *et al.* demonstrated that proteins and microRNAs from exosomes in CLL patients trigger an inflammatory response within the recipient stromal cells, akin to CAFs^[63]^, prompting heightened proliferation, migration, and the release of inflammatory cytokines, thereby fostering an immunosuppressive TME^[63]^. Burger *et al.* further demonstrated that CLL cells are shielded by blood-derived NLCs, which differentiate into large fibroblast-like cells expressing stromal cell markers and stromal cell-derived factor-1 (SDF-1). CLL cells attach to these NLCs and downregulate receptors for SDF-1 (CXCR4) and are protected from apoptosis^[65]^. Additionally, CLL cells also induce MDSCs converted from healthy monocytes, which secrete indoleamine 2.3-dioxygenase (IDO) and limit T-cell function and proliferation, and promote Tregs^[66]^. IDO has also been found to inhibit CAR T-cells, but its expression seems to be downregulated with lymphodepleting chemotherapy^[68]^. Studies have also demonstrated that the TME plays a crucial role in determining CAR T-response. Extracellular vesicles, specifically PD-L1+ vesicles, in patients with CLL induce CAR T-cell exhaustion^[67]^. Additionally, immunosuppressive myeloid cells not only have a critical role in the suppression of CAR T-cell effector functions, but can also potentiate CRS and neurotoxicity^[64]^. The development of newer generations of CAR T-cells incorporates strategies to overcome the immunosuppressive TME.

## STRATEGIES FOR OVERCOMING RESISTANCE

**Figure 4:**
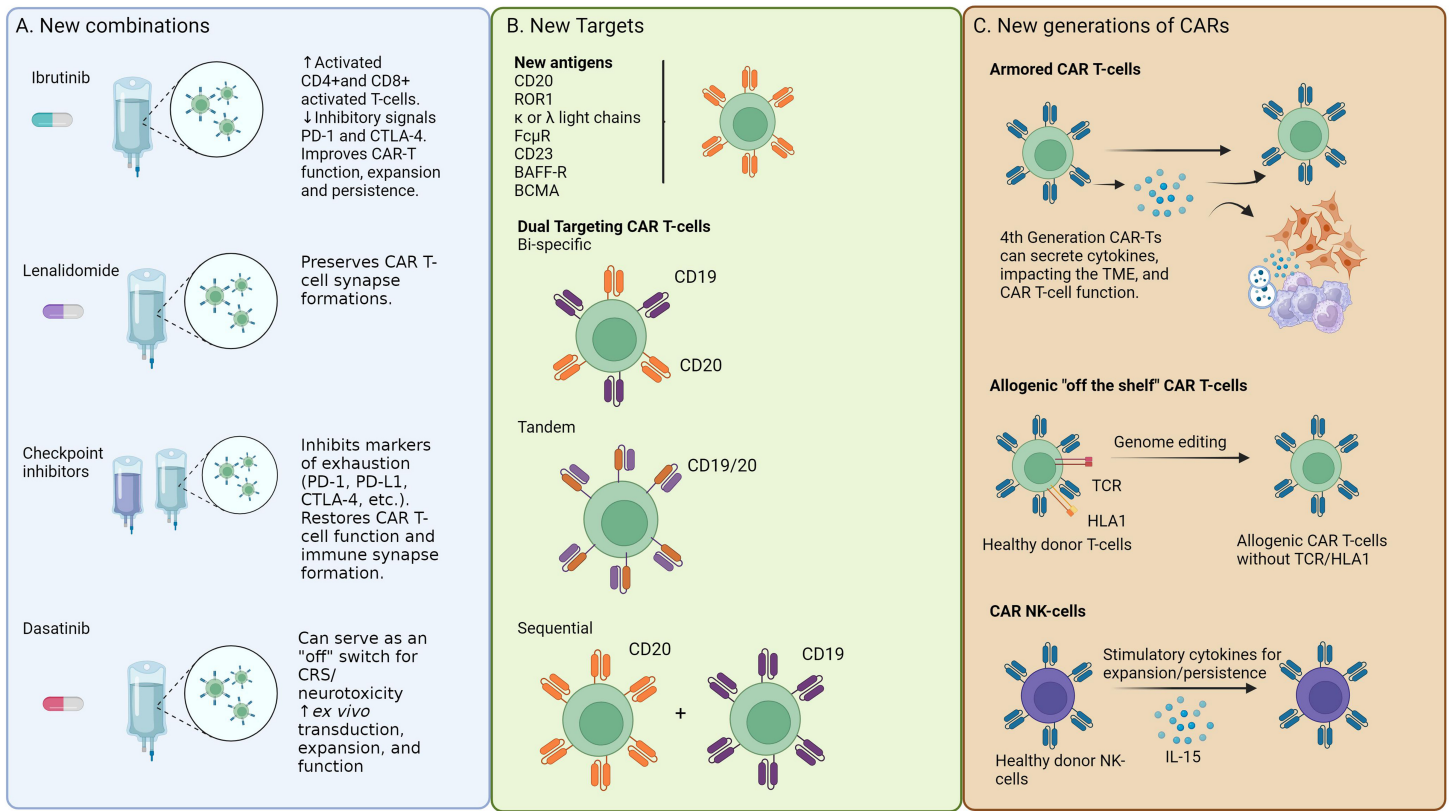
Strategies to overcome resistance. (A) New combinations with CAR T-cell infusions include ibrutinib, lenalidomide, checkpoint inhibitors, and dasatinib with promising mechanisms of improving CAR T-cell function, expansion, durability, and safety; (B) Novel targets including new antigens of interest, and dual targeting CAR T-cell methods. Dual targeting methods include bispecific CAR T-cells, which include 2 different CARs targeting 2 antigens per T-cell, tandem CAR T-cells with 2 antigen targets on each CAR, and sequential including 2 CAR T-cells targeting different antigens given together or sequentially; (C) New generations of CAR T-cells such as “armored” CARs that can express a protein such as a cytokine that can help regulate the CAR T-cell and impact the TME. Allogenic CAR T-cells utilize healthy donor T-cells for an “off the shelf” option. They have the TCR and HLA1 receptors deleted to prevent complications with graft-versus-host-disease. CAR NK-cells can also utilize healthy donor cells and do not need to have TCR/HLA modification; however, they need additional stimulatory molecules to improve expansion and persistence. CAR: Chimeric antigen receptor; TME: tumor microenvironment; TCR: T-cell receptor; HLA1: human leukocyte antigen type 1; NK: natural killer; PD-1: programmed death-1; CTLA-4: cytotoxic T-lymphocyte-associated protein 4; PD-L1: programmed death ligand-1; CRS: cytokine release syndrome; ROR1: receptor tyrosine kinase like orphan receptor 1; FcµR: Fcµ receptor; BAFF-R: B-cell activating factor receptor; BCMA: B-cell maturation antigen.

Despite the notable achievements observed with CAR T-cell therapy, ongoing studies are exploring diverse approaches to enhance their anti-tumor effects. These include combining other agents with CAR T-cells, developing novel CAR T-cell constructs targeting different antigens, using dual-targeting CAR T-cells, and incorporating “armored” CAR T-cells to modify the immunosuppressive TME. Furthermore, allogeneic CAR T- and natural killer (NK)-cells are being pursued to address the challenge of poor T-cell fitness associated with CLL. Figure 4 provides an overview of these strategies. A recent comprehensive list of all ongoing CAR trials in CLL was recently published^[69]^.

### Combination therapies with CAR T-cells

#### Ibrutinib

The combination of ibrutinib and CAR T-cell therapy has emerged as an exciting area of research, offering potential improvements in both efficacy and safety^[70,71]^. Ibrutinib inhibits both BTK, inducing apoptosis in B-cells, and IL-2 inducible T-cell kinase (ITK)^[72]^. This dual effect leads to markedly increased activated CD4+ and CD8+ T-cells with enhanced persistence, not observed with other selective BTK inhibitors, like acalabrutinib. BTK inhibitors also reduce PD-1 and CTLA-4 expression, leading to dampening of the immunosuppressive signals in CLL^[72]^.

Multiple clinical trials in CLL have demonstrated synergy with the combination of ibrutinib and CAR T-cell therapy. In a study of 19 heavily pretreated patients, 89% of whom had high-risk cytogenetics, ibrutinib was administered 2 weeks prior to leukapheresis and continued for 3 months post-CAR T-cell infusion. They found an ORR of 86%, with 59% achieving undetectable MRD in the marrow by IGH sequencing. They also observed lower CRS severity compared to historical rates and reduced levels of CRS-associated cytokines^[7]^. However, they did report 1 death from likely cardiac arrhythmia in the ibrutinib cohort. In a study by Gill *et al.*, CD19 CAR T-cell therapy was added to 19 patients who did not achieve a CR after at least 6 months of ibrutinib therapy. The 3-month CR rate was 44%, with a 72% undetectable MRD rate at 12 months^[7,73]^. Eighteen subjects had CRS, mostly grades 1-2, while 5 developed neurotoxicity (4 grades 1-2, and 1 grade 5). One patient died of cardiac arrest with concurrent grade 4 CRS and neurotoxicity. The TRANSCEND CLL 004 Phase 1/2 trial also studied a cohort of patients using liso-cel in combination with ibrutinib^[74]^. Preliminary data from this cohort, comprising 19 patients refractory to ibrutinib, showed a 95% ORR, with 47% achieving a CR. Additionally, 89% of patients achieved undetectable MRD in blood by flow cytometry and 79% in the bone marrow^[75]^. Correlative studies revealed improved CAR-T cell expansion and reduction of inflammation-associated genes including IL-6^[76]^. T-cell exhaustion genes were significantly lower in the combination arm, correlating with improved PFS^[76]^. Currently, a Phase I/II study of the rapidly manufactured (< 2 days) autologous CD19 CAR T-cell product (known as rapcabtagene autoleucel) in combination with ibrutinib in r/r CLL/SLL (NCT03960840) is ongoing.

#### Lenalidomide

Lenalidomide, an immunomodulatory agent, is also under investigation as a combinational agent. Studies show that lenalidomide can repair defective synapse formation between T-cells and antigens in CLL patients^[52]^. A recent preclinical study evaluating lenalidomide combined with CD23-specific CAR-T cells demonstrated that lenalidomide preserved CAR T-cell immune synapses and enhanced migration to leukemic sites, delaying disease progression^[77]^. Additionally, co-administration of lenalidomide with CD19 and CD20 CAR T-cells *in vivo* results in diminished tumor expansion, infiltration of cytotoxic T-cells into the tumor, and interferon-γ production^[78]^.

#### Checkpoint inhibitors

The application of checkpoint inhibitors (CPIs) in CLL has led to disappointing results, with a response only seen in Richter transformation, and only in less than half of cases^[79]^. Nevertheless, given the increased expression of exhaustion markers on CLL CAR T-cells, there is a strong rationale for adding CPIs as combinational agents. In CLL-like mouse models, PD-L1 blockade helped to restore aberrant and exhausted T-cell phenotypes as well as CD8 T-cell cytotoxicity and immune synapse formation^[80]^. Preliminary safety studies combining CPIs with the three approved CD19 CAR T-cell agents in LBCLs have all demonstrated favorable safety profiles with signals of improved efficacy^[81-83]^.

#### Dasatinib

Dasatinib is a tyrosine kinase inhibitor that has gained interest for its unique ability to serve as an “off switch” for CRS developed from CAR T-cell therapy^[84]^. It interferes with lymphocyte-specific protein tyrosine kinase (LCK) and, through a series of events, diminishes CAR CD3ζ activation and downstream gene expression^[84]^. In mouse models, it was shown to turn off both CD8+ and CD4+ CAR T-cells immediately and could be reversed upon discontinuation of dasatinib without affecting T-cell viability. Interestingly, dasatinib can also enhance CAR T-cell transduction, expansion, and possible function when added during the manufacturing process^[85,86]^.

Overall, these studies suggest that the synergistic effect of CAR T-cell therapy with other therapies, most notably ibrutinib, may enhance efficacy, reduce immune suppression, and improve safety. Longer term data in larger studies will be needed to fully understand their applications in CLL.

### New targets and dual targeting CAR T-cells

In addition to combinational strategies, innovative CAR T-cell constructs are under investigation, including the targeting of novel antigens to combat antigen escape. In addition, dual-targeting CAR T-cells such as bispecific CARs, tandem CARs, and sequential CAR T-cell therapy are of great interest [Figure 4]^[87]^. Triple-targeting CAR T-cells are also being investigated. Careful antigen selection is crucial to minimize off-target binding and adverse effects. Promising antigen targets include CD20, receptor tyrosine kinase-like orphan receptor 1 (ROR1), kappa (κ) or lambda (λ) light chains, Fcµ receptor (FcµR), CD23, B-cell activating factor-receptor (BAFF-R), and B-cell maturation antigen (BCMA).

#### CD20

CD20, found in over 90% of B-cell lymphomas, remains consistently expressed even after CD20-directed therapy and is therefore a common target for lymphoma treatment^[88]^. Nevertheless, akin to the CD19 target, it raises concerns regarding B-cell aplasia and agranulocytosis^[88]^. Early studies with third-generation CD20-targeting CAR T-cells showed promising results in low-grade B-cell lymphomas, with some patients still in remission at 24 months^[89]^. However, challenges arise in CLL due to modest CD20 expression and often exposure to prior CD20-directed therapies^[90]^. Nevertheless, CD20-targeted CAR T-cells can exhibit detectability and CAR T-cell activation even in cells with low antigen expression, holding promise for patients with CLL who have shown resistance to CD20 monoclonal antibodies^[91]^.

Dual-targeting CAR T-cells, which simultaneously target CD19 and CD20, are also being explored. Innovations in CAR T-cell development have given rise to bispecific^[92]^ and tandem^[93]^ CAR designs, demonstrating their ability to induce cell killing against either CD19- or CD20-positive cells, helping prevent antigen escape as the CAR remains primed for activation by the other antigen if one is downregulated or lost. In a study of patients with r/r NHL, including three with CLL, dual CD19-CD20 CAR T-cells demonstrated an 88% ORR and a 64% CR rate. Among the CLL patients, two achieved CR, while one achieved PR. The duration of response for those in CR has yet to be determined^[94]^. However, further research is needed to thoroughly delineate the efficacy and safety profiles of these innovative CAR products in CLL. Several dual CAR T-cell trials are currently under investigation, with a notable trial on CD19-CD20-CD22 trispecific CAR T-cells (NCT05418088).

#### ROR1

ROR1 is another interesting antigen found predominantly in CLL and MCL cells^[95]^. While ROR1 is highly expressed in undifferentiated embryonic stem cells, it is not found in other adult tissue types except for a low expression in adipose tissue^[96]^. Additionally, higher levels of ROR1 on CLL cells are associated with more aggressive disease, and its expression remains stable across progression and treatment^[97]^. Therefore, it presents an attractive prospect for achieving highly targeted effects, thereby mitigating adverse events and reducing immunosuppression. Hudecek *et al.* reported the first ROR1-targeted CAR-T cell with cytotoxicity in CLL and MCL cells^[96]^. More recently, Prussak *et al.* reported on a ROR1-specific CAR T-cell that effectively and selectively killed ROR1-containing lymphoid cancer cells in mice^[98]^.

#### κ/λ

CLL cells, like plasma cells in multiple myeloma, frequently exhibit clonal expression of either the κ or λ light chains, offering a potential targeted intervention that leaves half of normal B-cells. Both κ and λ -specific CAR T-cells have emerged from pre-clinical studies as promising candidates for treating CLL^[99]^. However, in an early trial involving 16 patients with NHL and multiple myeloma, κ-specific CAR T-cell therapy yielded only a low response rate of 33%^[100]^. Regrettably, the two CLL patients in the study did not respond. This could be due to insufficient lymphodepleting therapy during the trial^[100]^. Presently, two single-center Phase 1 trials are evaluating κ-targeting CAR T-cells in r/r NHL and CLL (NCT04223765 & NCT00881920).

#### BAFF-R and BCMA

Two other potential targets in CLL cells are BAFF-R and BCMA, both activated by the protein BAFF, which is expressed by immunosuppressive cells in the TME in CLL and promotes disease progression and chemotherapy resistance^[101]^. BAFF-R signaling is essential for B-cell survival and is expressed on malignant B-cells. BAFF-R CAR T-cells have demonstrated anti-tumor activity in NHL including CLL in preclinical models^[102,103]^ and can overcome CD19 antigen loss in B-cell malignancies^[104]^. There are currently ongoing Phase 1 studies of BAFF-R targeting CAR T-cells in r/r B-ALL (NCT04690595) and B-NHL (NCT05370430). A dual BAFF-R/CD19 CAR T-cell is under development to further curb antigen evasion^[105]^. Another promising target is BCMA, which is also elevated in CLL patients and correlates with more aggressive disease^[106]^. BCMA is typically prevalent in advanced-stage B-cells and plasma cells and has already been successfully targeted in multiple myeloma, leading to the FDA approval of two BCMA-CAR T-cell therapies: idecabtagene vicleucel (ide-cel)^[107]^ and ciltacabtagene autoleucel (cilta-cel)^[108]^. There is an ongoing trial using CD19-BCMA dual CAR T-cell therapy in combination with Dasatinib in patients with r/r B-ALL, NHL including CLL, and multiple myeloma (NCT04603872).

#### Fcµ receptor and CD23

Another studied target is FcµR, also recognized as TOSO or FAIM3^[109]^. This receptor is consistently highly expressed in CLL cells and minimally expressed in healthy B-cells and other tissues. Mouse studies have shown promising results with FcµR CAR T-cells effectively eliminating autologous CLL cells, while preserving healthy B-cells^[109]^. CD23 CAR T-cells have also shown promising results in preclinical models when combined with lenalidomide^[77]^. However, comprehensive data are needed to fully understand the implications of these and other targets under investigation in CLL.

### Altering the immunosuppressive TME, and the rise of the next generation of autologous CAR T-cells

Modifying the immunosuppressive TME in CLL could greatly enhance the CAR T-cells function. Augmenting pro-inflammatory cytokines is one approach. Early attempts to enhance adoptive immunotherapy were based on *ex vivo* culture and exogenous administration of cytokines IL-2 and IL-15, which augmented T-cell activity; however, these strategies were also associated with worse systemic toxicity^[110]^. In order to limit toxicity but still get the immunomodulatory effects of IL-2, a new strategy combines SYNCAR-001, a CD19-targeting CAR T-cell that expresses an engineered IL-2 receptor, and STK-009, a pegylated IL-2 cytokine that selectively binds only CAR T-cells^[111]^. This combination is being studied in a Phase 1 trial for CD19+ hematological malignancies, including CLL (NCT05665062). Additionally, exciting 4th generation armored CAR-T cells show promise in preclinical studies^[20]^, necessitating further clinical assessment in CLL.

### Allogenic CARs provide an exciting “off the shelf” alternative

Preserving high-quality T-cells suitable for CAR T-cell manufacturing in CLL patients is challenging due to T-cell exhaustion from prolonged antigen exposure and usually years of prior treatments. Furthermore, CAR T-cell manufacturing may take weeks, posing obstacles for rapidly progressive disease. Developing “off the shelf” allogenic CAR T-cells from healthy donor cells bypasses these challenges and dissolves the risk of leukemic cell contamination. Initially, there were concerns about graft-versus-host disease (GVHD) using allogenic CAR T-cells; however, gene-editing technologies such as zinc-finger nuclease, transcription activator-like effector nuclease (TALEN), or clustered regularly interspaced short palindromic repeats (CRISPRs) offer solutions by disrupting the *TCR* genes and reducing GVHD risk^[112]^.

Brudno *et al.* documented an early instance of allogeneic CAR T-cells in humans. In their study, 20 patients with B-cell malignancies, including 5 CLL patients who relapsed after an allogenic transplant, received CD19 allogeneic CAR T-cells. Only one CLL patient achieved a sustained CR that persisted beyond 30 months of follow-up. The safety profile was similar to autologous CAR T-cell studies, with no instances of GVHD^[113]^. The CD19 allogeneic CAR T-cell FT819, which is an induced pluripotent stem cell (iPSC) line that can serve as a renewable source for mass production, in combination with IL-2^[114]^, is currently under study in a phase 1 trial throughout the US (NCT04629729). In another promising Phase 1 trial, called ARDENT, a new “hypoimmune” CD19-directed allogeneic CAR T-cell therapy was designed to reduce immune recognition by depletion of HLA I/II to prevent adaptive immune rejection and overexpression of CD47 to thwart innate immune rejection^[115]^. This trial is currently recruiting patients with r/r NHL and CLL within Australia and the US (NCT05878184).

CAR NK cells have similar components to CAR T-cells, namely an antigen (scFvs)-recognition domain, an activation domain (usually CD3ζ), and costimulatory domains (CD18, 4-1BB, or 2B4)^[116]^. NK cells offer advantages such as HLA-independent cytotoxicity and no rejection reactions, making them suitable for allogeneic products sourced from healthy donor blood or cord blood^[117,118]^. Challenges in using NK CARs include difficulties with transfection, expansion, and durability, which have been addressed through various methods^[119]^. For instance, Liu *et al.* successfully engineered cord blood (CB) CAR NK-cells with expression of IL-15 and an inducible caspase 9 safety switch^[117]^. In a phase 1 study, this CB-CAR NK cell construct showed promising results, yielding a CR in 3 of the 5 patients in the study with CLL^[120]^. The expansion phase of this study, called the ANCHOR trial, is ongoing (NCT03774654). These readily available CAR options have the potential to emerge as more convenient alternatives to autologous CAR T-cells. Numerous other trials are underway, investigating innovative new CAR T and NK-cell products for the treatment of NHL, including CLL.

## CONCLUSIONS

CAR T-cell therapy presents a compelling approach for addressing the treatment challenges in high-risk and multiply refractory patients with CLL. However, challenges persist in CLL due to underlying T-cell defects and lower response rates. Nevertheless, recent trials have shown promising results, leading to the first FDA approval of liso-cel for CLL patients who have failed BTK and BCL2 inhibitors. This “one and done” therapeutic approach holds the potential to offer a long-term remission for some patients, yielding prolonged responses exceeding a decade, as evidenced in some cases. While resistance mechanisms, such as antigen escape and T-cell exhaustion, pose hurdles, ongoing research aims to overcome these barriers through combination therapies, novel targets, and strategies to alter the immunosuppressive TME. Ongoing investigations into combinational agents and the development of the next generation of CAR constructs including allogenic and armored versions, offer a promising trajectory, heralding a new therapeutic era in the realm of CLL treatment.

## DECLARATIONS

### Copyright

© The Author(s) 2024.
